# Hemodynamic management of critically ill burn patients: an international survey

**DOI:** 10.1186/s13054-018-2129-3

**Published:** 2018-08-17

**Authors:** Sabri Soussi, Mette M. Berger, Kirsten Colpaert, Martin W. Dünser, Anne Berit Guttormsen, Nicole P. Juffermans, Paul Knape, Guniz Koksal, Athina Lavrentieva, Thomas Leclerc, José A. Lorente, Ignacio Martin-Loeches, Philipp Metnitz, Olivier Pantet, Paolo Pelosi, Anne-Françoise Rousseau, Folke Sjöberg, Matthieu Legrand, for the ESICM Burn ICU working group

**Affiliations:** 1 Department of Anesthesiology and Critical Care and Burn Unit, AP-HP, Hôpital Saint-Louis, 1 Avenue Claude Vellefaux, 75010 Paris, France; 2 Service of Adult Intensive Care Medicine and Burns, University Hospital, 1011 Lausanne, Switzerland; 3 Department of Intensive Care and Burns, Ghent University Hospital, Ghent, Belgium; 4 Department of Anesthesiology and Intensive Care Medicine, Kepler University Hospital and Johannes Kepler University Linz, Linz, Austria; 5 Department of Anaesthesiology and Intensive Care, Haukeland University Hospital and University of Bergen, Bergen, Norway; 6 Department of Intensive Care Medicine, Academic Medical Center, Laboratory of Experimental Intensive Care and Anesthesiology (LEICA), Amsterdam, The Netherlands; 7 Department of Anesthesiology, Red Cross Hospital, Beverwijk, The Netherlands; 8 Department of Anesthesiology and Reanimation, Cerrahpasa Medical School, Istanbul University, Istanbul, Turkey; 9 Burn Unit, Papanikolaou Hospital, Thessaloniki, Greece; 10 Burn Centre, Percy Military Hospital, Clamart, France; 11 Critical Care and Burn Unit, Hospital Universitario de Getafe, CIBER de Enfermedades Respiratorias, Universidad Europea de Madrid, Madrid, Spain; 12 Department of Clinical Medicine, Trinity College, Welcome Trust-HRB Clinical Research Facility, St James Hospital, Dublin, Ireland; 13 Department of General Anaesthesiology, Emergency and Intensive Care Medicine, LKH - University Hospital of Graz, Medical University of Graz, Graz, Austria; 14 Department of Surgical Sciences and Integrated Diagnostics, San Martino Policlinico Hospital, IRCCS for Oncology, University of Genoa, Genoa, Italy; 15 Burn Centre and Intensive Care Department, University Hospital of Liège, Liège, Belgium; 16 Departments of Hand, Plastic and Burns and Intensive Care, Linköping University Hospital, Linköping University, 581 85 Linkoping, Sweden; 17 Department of Anesthesiology and Critical Care and Burn Unit, AP-HP, Hôpital Saint-Louis, Hôpital Lariboisière, UMR Institut National de la Santé et de la Recherche Médicale (INSERM) 942, Université Paris Diderot, F-75475 Paris, France

Fluid resuscitation is a cornerstone of the initial management of severely burned patients with the dual purpose of avoiding both under- and over-resuscitation [1–3]. There is a lack of consensus regarding the ideal amount and type of fluid and vasopressor use during initial resuscitation in this population [4, 5].

This international survey focuses on the current practices regarding hemodynamic management of severely burned adult patients (total body surface burn area (TBSA) > 20%, with mechanical ventilation) in the early phase after injury.

The study was designed as an electronic survey addressed to intensive care unit (ICU) physicians. Experts of the European Society of Intensive Care Medicine (ESICM) Burn ICU working group were invited to review the original survey. The final questionnaire (32 questions) is provided in Additional file 1. A link to an electronic questionnaire was sent to all ESICM members (with reminding emails on a bimonthly frequency) and was posted on the ESICM website. The link was active between 31 August and 18 October 2017.

There were 173 total respondents to the questionnaire. The respondents were from 58 different countries (72% were high-income countries) with most in Europe (62%). The background of the respondents was mainly intensive care (61%) and anesthesiology (31%). Most of the respondents (61%) declared working in a mixed ICU, and 60% of the responders worked in centers with less than 50 adult burn patients admitted annually. Additional file 2 summarizes the difference in participant responses between burn centers and nonspecialized centers. In 76% of the cases, a local protocol for fluid resuscitation was used. The Parkland formula (4 ml/kg/%TBSA) is used to start volume therapy on admission by 54% of the responders. In the first 48 h, the five most frequently used parameters to guide volume therapy are represented in Fig. 1a. Fifty five % of the respondents declared monitoring cardiac output and 65% among them use echocardiography. Techniques used to monitor cardiac output continuously are presented in Fig. 1b. The most commonly used crystalloid and colloid were respectively Ringer Lactate and albumin 20%. Triggers to initiate colloid infusion are presented in Fig. 1c. While considering other strategies to reduce fluid requirements, 80% of responders consider early norepinephrine administration (Fig. 1d).

**Fig. 1:**
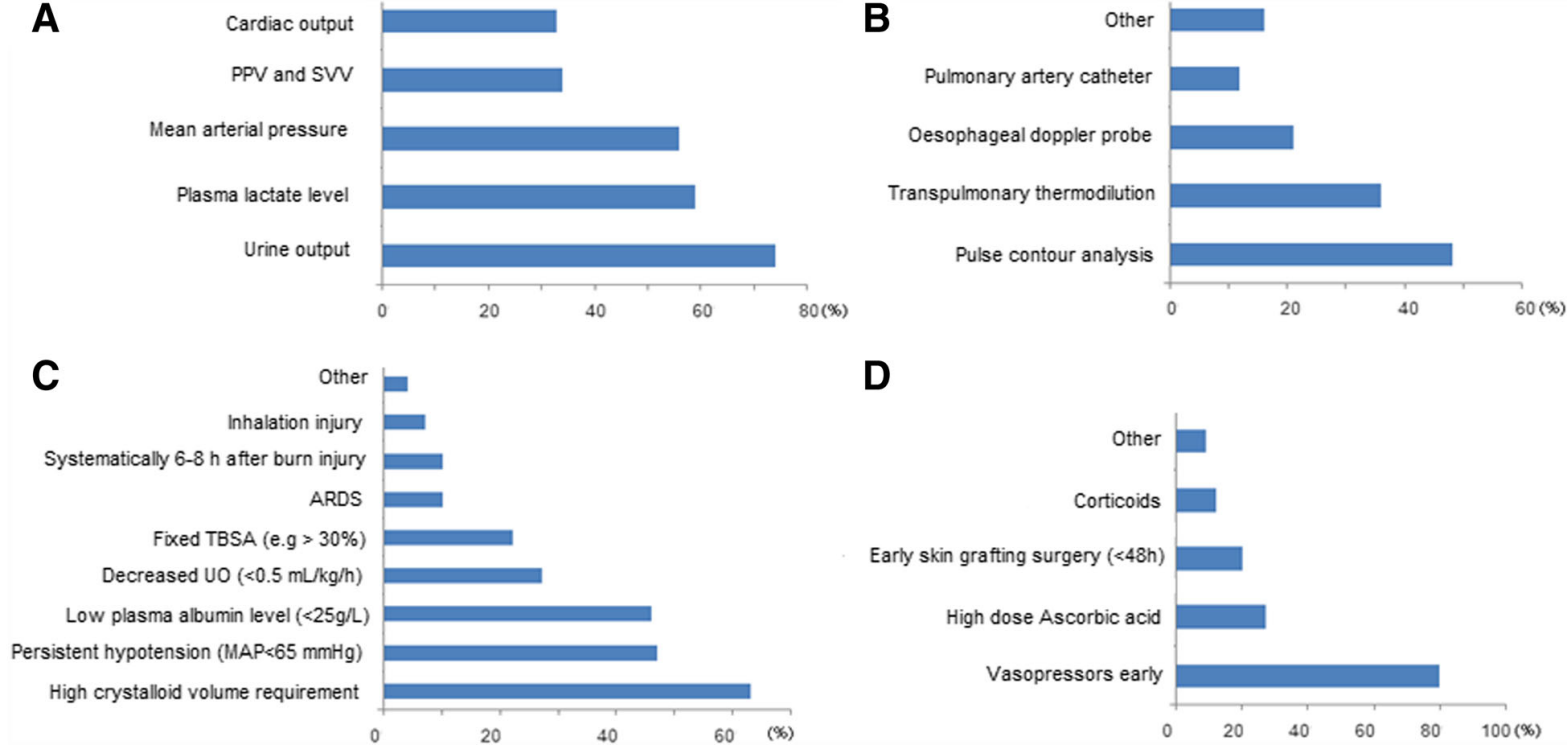
**a** The five most frequently used parameters to guide volume therapy in severely burned patients. **b** Techniques used to monitor cardiac output continuously. **c** Triggers to initiate colloid infusion. **d** Adjunctive therapies to reduce initial volume administration. ARDS acute respiratory distress syndrome, MAP mean arterial pressure, PPV pulse pressure variation, SVV stroke volume variation, TBSA total body surface burn area, UO urine output

The results of this international survey highlight the use of albumin (> 60%) and vasopressors (80%) during the early resuscitation phase. Heterogeneous results were reported regarding monitoring strategies, early vasopressors, and albumin use between burn centers and nonspecialized centers. Large clinical trials should be initiated in the near future to determine optimal strategies to treat burn-related shock.

## Additional files

Additional file 1: Survey questions. (PDF 131 kb)

Additional file 2: Comparison of participant responses between burn centers and nonspecialized centers. CO cardiac output, *n* number of respondents per group. The results are reported as numbers and percentages (%). The chi^2^ and Fischer tests were used as appropriate (*p* < 0.05). (PDF 155 kb)

## Abbreviations

ESICM: European Society of Intensive Care Medicine
ICU: Intensive care unit
TBSA: Total body surface burn area
