# Supplementary material for: Hemodynamic management of critically ill burn patients: an international survey
Source: Crit Care. 2018 Aug 17;22:194. doi: 10.1186/s13054-018-2129-3 (PMC6097223; doi:10.1186/s13054-018-2129-3)
Supplement: Supplementary file 1 — Survey questions. (PDF 131 kb) [file 13054_2018_2129_MOESM1_ESM.pdf]

Hemodynamic management of critically ill burn patients:

An International survey

1) Characteristics of your hospital:

□ University hospital

□ Public community hospital

□ Private hospital

□ Exclusive burn center

□ Other, please specify: …………………..

2) Characteristics of your intensive care unit (ICU):

□ Medical ICU

□ Surgical ICU

□ Mixed ICU

□ Intensive care burn unit

3) Annual number of burns patients treated in the ICU (approximatively):

□ ≥ 500 patients

□ 200-500 patients

□ 100-200 patients

□ 50-100 patients

□ < 50 patients

4) Total number of ICU beds (do not include medium care beds):

□ > 20 beds

□ 16-20 beds

□ 11-15 beds

□ 6-10 beds

□ ≤ 5 beds

5) **Number of ICU Beds devoted to burns patients (do not include medium care beds):**

□ > 20 beds

□ 16-20 beds

□ 11-15 beds

□ 6-10 beds

□ ≤ 5 beds

6) Your burned patients are:

□ Only Adults

□ Both children and adults

□ Only children

7) Your main specialty (specialties) is (are): (More than one answer is possible)

□ Intensive care

□ Anesthesiology

□ Surgery

□ Other, please specify: …………………..

8) In which country do you work?

………………………………….

9) Experience in treating burn patients

□ < 2 years

□ Between 2 and 5 years

□ >5 years

10) Do you work full time with burns patients?

□ Yes □ No

11) Do you use a protocol for fluid resuscitation for burns patients in your unit?

□ Yes □ No

12) Which formula do you use to start volume therapy on admission in your burn patients?

□ Classical Parkland formula (4ml/Kg/% TBSA)

□ Modified Parkland formula (2ml/Kg/% TBSA)

□ Other formula, please specify: ……………………………

□ I do not use any formula

13) Do you routinely increase fluid volume resuscitation in the case of inhalation injury?

□ Yes □ No

14) What parameters do you usually use to guide your volume therapy in severely burn patients in your unit? (In descending order of importance n°…..) (If you do not use any of them, leave it unchecked)

□ Urine output (n°……)

□ Mean arterial pressure (MAP) (n°……)

□ Central venous pressure/ Right atrial pressure (n°……)

□ Pulmonary capillary wedge pressure (PCWP) (n°……)

□ Global end diastolic volume (GEDV) / Intrathoracic blood volume (ITBV) (n°……)

□ Cardiac output (n°……)

□ Plasma lactate levels (n°……)

□ Plasma base deficit (n°……)

□ Central or mixed venous oximetry (ScvO2 or SvO2) (n°……)

□ Venous-to-arterial carbon dioxide difference (PCO2 gap) (n°……)

□ Pulse pressure variation (PPV) / Stroke volume variation (SVV) (n°……)

□ Echocardiography based parameters (n°……)

□ Skin mottling (n°……)

□ Extravascular lung water (EVLW) (n°……)

□ Other, please specify: …………………………… (n°……)

15) Do you usually (> 50% of cases) monitor cardiac output in the first 48 hours after admission in your severe burn patients?

□ Yes □ No

16) If yes, which technique do you use to monitor cardiac output continuously? (More than one answer is possible)

□ Oesophageal doppler probe

□ Transpulmonary thermodilution

□ Pulse contour analysis

□ Pulmonary artery catheter

□ Other, please specify: ……………………………

17) Do you use echocardiography to guide fluid resuscitation in your severe burn patients?

□ Yes **□ No**

18) If yes, do you use: (More than one answer is possible)

□ Transthoracic Echocardiography □ Transesophageal Echocardiography □ Both

19) Which crystalloids do you use for the primary resuscitation in the first 48 hours?

**NaCl 0.9%**	**1) Almost never □**	**2) Rarely □**	**3) Sometimes □**	**4) Frequently □**	**5) Almost always □**

**Ringer's Lactate**	**1) Almost never □**	**2) Rarely □**	**3) Sometimes □**	**4) Frequently □**	**5) Almost always □**
**Plasma-Lyte®**	**1) Almost never □**	**2) Rarely □**	**3) Sometimes □**	**4) Frequently □**	**5) Almost always □**
**Isofundine®**	**1) Almost never □**	**2) Rarely □**	**3) Sometimes □**	**4) Frequently □**	**5) Almost always □**
**Other, please specify: ……………………**	**1) Almost never □**	**2) Rarely □**	**3) Sometimes □**	**4) Frequently □**	**5) Almost always □**

Almost never (0 to 20 %), 2) Rarely (21 to 40%), 3) Sometimes (41 to 60%), 4) Frequently (61 to 80%), 5) Almost always (81 to100%)

20) Do you use colloids?

**1) Almost never □**	**2) Rarely □**	**3) Sometimes □**	**4) Frequently □**	**5) Almost always □**

Almost never (0 to 20 %), 2) Rarely (21 to 40%), 3) Sometimes (41 to 60%), 4) Frequently (61 to 80%), 5) Almost always (81 to100%)

21)Which colloids do you use for the primary resuscitation in the first 48 hours?

**HES**	**1) Almost never □**	**2) Rarely □**	**3) Sometimes □**	**4) Frequently □**	**5) Almost always □**

**Gelatins**	**1) Almost never □**	**2) Rarely □**	**3) Sometimes □**	**4) Frequently □**	**5) Almost always □**
**Dextrans**	**1) Almost never □**	**2) Rarely □**	**3) Sometimes □**	**4) Frequently □**	**5) Almost always □**
**Albumin 20%**	**1) Almost never □**	**2) Rarely □**	**3) Sometimes □**	**4) Frequently □**	**5) Almost always □**
**Albumin 5%**	**1) Almost never □**	**2) Rarely □**	**3) Sometimes □**	**4) Frequently □**	**5) Almost always □**
**Fresh frozen plasma**	**1) Almost never □**	**2) Rarely □**	**3) Sometimes □**	**4) Frequently □**	**5) Almost always □**

HES= Hydroxyethylstarches

Almost never (0 to 20 %), 2) Rarely (21 to 40%), 3) Sometimes (41 to 60%), 4) Frequently (61 to 80%), 5) Almost always (81 to100%)

22) If you use colloids, how long do you wait before colloid infusion initiation?

□ 6-8 hours after burn injury

□ 12 hours after burn injury

□ 24 hours after burn injury

23) If you use colloids, what are the triggers to initiate colloid infusion? (More than one answer is possible)

□ Systematically 6-8 hours after burn injury

□ Fixed total burned surface area (e.g. TBSA > 30%)

□ Persistent hypotension (MAP< 65 mmHg)

□ High cristalloid volume requirement

□ Acute respiratory distress syndrome (ARDS)

□ Low plasma albumin levels (< 25 g/L)

□ Inhalation injury

□ Decreased urine output (< 0.5 ml/Kg/h)

□ Other: ………………………………………………

24) Do you use adjunctive therapies to reduce volume administration in the first 48 hours after burn injury? (More than one answer is possible)

□ I use vasopressors early

□ I use high dose Ascorbic acid (Vitamin C)

□ I use early skin grafting surgery (<48 hours)

□ I use corticoids

□ Other, please specify: ……………………………

26) What is your first line vasopressor in the initial treatment of your severe burn patients?

□ Dopamine

□ Norepinephrine

□ Epinephrine

□ Phenylephrine

□ Vasopressin

□ Metaraminol

□ Other: ……………………………

27) Which MAP do you generally target in a young severely burned patient without comorbidities?

□ 60 mmHg

□ 65 mmHg

□ 70 mmHg

□ 75 mmHg

28) Which Cardiac index (CI) do you generally target?

□ 2-2.5 L/min/m²

□ 2.5-3 L/min/m²

□ 3-3.5 L/min/m²

□ > 3.5 L/min/m²

□ I consider CI variations rather than a specific number

30) Do you use vasodilators in the first 24 hours after burn injury?

1) Almost never □	2) Rarely □	3) Sometimes □	4) Frequently □	5) Almost always □

Almost never (0 to 20 %), 2) Rarely (21 to 40%), 3) Sometimes (41 to 60%), 4) Frequently (61 to 80%), 5) Almost always (81 to100%)

31) **If yes, which vasodilators do you use for the primary resuscitation in the first 24 hours?**

□ Nitroglycerin

□ Dobutamine

□ Enoximone

□ Prostacyclin

□ pentoxifylline,

□ Other: ……………………………
