# Supplementary material for: Hemodynamic management of critically ill burn patients: an international survey
Source: Crit Care. 2018 Aug 17;22:194. doi: 10.1186/s13054-018-2129-3 (PMC6097223; doi:10.1186/s13054-018-2129-3)
Supplement: Supplementary file 2 — Comparison of participant responses between burn centers and nonspecialized centers. CO cardiac output, n number of respondents per group. The results are reported as numbers and percentages (%). The chi2 and Fischer tests were used as appropriate (p < 0.05). (PDF 155 kb) [file 13054_2018_2129_MOESM2_ESM.pdf]

Additional file 2 Comparison of participants' responses between burn centers and non-specialized centers

	Respondents of non-specialized centers	Respondents of burn centers	p
**Use of classical Parkland, n (%)**	52/89 (58%)	18/37 (48%)	0.31
**Usually monitor CO (>50%) in the first 48 hrs, n (%)**	43/130 (33%)	27/41 (65%)	0.001
**Frequently or almost always use colloids, n (%)**	15/80 (19%)	22/37 (59%)	<0.001
**Frequently or almost always use Albumin, n (%)**	39/76 (51%)	26/36 (72%)	0.03
**Early use of vasopressors, n (%)**	68/130 (52%)	25/41 (61%)	0.001

CO cardiac output, *n* number of respondents per group. The results are reported as numbers and percentages (%). The chi2 and Fischer tests were used as appropriate ($p<0.05$).
